# Predictive Value of Max’s Giant Associated Protein Mutation in Outcomes of Lung Adenocarcinoma Patients Treated With Immune Checkpoint Inhibitors

**DOI:** 10.3389/fcell.2021.728647

**Published:** 2021-10-18

**Authors:** Yan Qu, Chao Wang, Lihui Liu, Sini Li, Xue Zhang, Zixiao Ma, Hua Bai, Jie Wang

**Affiliations:** State Key Laboratory of Molecular Oncology, Department of Medical Oncology, National Cancer Center/National Clinical Research Center for Cancer/Cancer Hospital, Chinese Academy of Medical Sciences and Peking Union Medical College, Beijing, China

**Keywords:** MGA mutation, pan-cancer, lung adenocarcinoma, immunotherapy, predictive biomarker

## Abstract

Treatment with immune checkpoint inhibitors (ICIs) has considerably improved prognosis in multiple cancers. However, regardless of PD-L1 expression and TMB, better predictive biomarkers are required to identify ICI-responsive patients. We analyzed a pan-cancer cohort as the discovery cohort to identify the role of Max’s giant associated protein (MGA) mutation in the outcome of ICI treatment in different types of cancers. A pooled lung adenocarcinoma (LUAD) cohort was considered as the validation cohort. Another two LUAD cohorts who received conventional treatment were included for prognostic analysis and mechanism exploration. In the discovery cohort, MGA mutation was a favorable survival biomarker for patients with LUAD than in those with other types of cancers. MGA mutation was positively correlated with the TMB score. The results of the validation cohort were consistent with those of the discovery cohort. Patients with MGA mutation in the TMB-low subgroup had longer survival. Two LUAD cohorts who received standard treatment showed that the MGA mutation was not a prognostic biomarker for standard treatment. Mechanically, we found that the co-mutant genes did not affect the prognostic role of MGA mutation. Gene-set enrichment analysis revealed that genes belonging to the immunodeficiency pathway were enriched in the MGA wild-type group in LUAD. Moreover, activated NK cells were more enriched in the MGA mutant LUAD group. In conclusion, our results demonstrated that MGA mutation was an independent predictive biomarker for ICI therapy. These results may provide a novel insight into identifying potential patients with LUAD for ICI therapy.

## Introduction

Immune checkpoint inhibitors (ICIs), consisting of T-lymphocyte antigen 4 (CTLA-4) inhibitor and programmed death-1 (PD-1)/programmed death ligand-1 (PD-L1) inhibitor, have attracted considerable attention because of their specific antitumor activity that has contributed to the prolonged survival of patients with multiple cancers (Ferris et al., 2016; Escudier et al., 2017; Wolchok et al., 2017; Wu et al., 2019), including those with non-small cell lung cancer (NSCLC). However, several patients could not benefit from ICI treatment. Hence, many studies focused on identifying a better predictive biomarker to choose patients who are most receptive to ICI treatment. Although tumor mutation burden (TMB) and PD-L1 expression are good predictive biomarkers of patient survival after ICI treatment (Rizvi et al., 2015; Hellmann et al., 2018), their predictive role is debatable. Some studies reported that up-regulated TMB or PD-L1 expression was correlated with the prolonged survival of patients after ICI treatment, whereas other studies revealed that the major pathologic response (MPR) was observed irrespective of PD-L1 expression and TMB (Yang et al., 2018; Addeo et al., 2019; Remon et al., 2020). Hence, exploring better predictive biomarkers for predicting the efficacy of ICIs is still necessary.

Max’s giant associated protein (MGA) encodes the Max-interacting protein belonging to the MYC pathway (Hurlin et al., 1999). MAX heterodimerizes with the MYC proto-oncogene and activates and mediates transcription activity of genes involved in controlling cell proliferation (Hurlin et al., 1999; Dang, 2012). MGA is a tumor suppressor gene (TSG) that binds to MAX and inhibits MYC-dependent tumor growth (Escudier et al., 2017). Recent studies revealed that MGA mutation occurs frequently in multiple malignant cancers (De Paoli et al., 2013; Cancer Genome, Atlas Research, and Network, 2014; Jo et al., 2016; Zhang et al., 2018), providing a new insight into tumorigenesis and heterogeneity.

Although many muted genes, including TP53, EGFR, STK11, KEAP1, MUC16, POLE, EPHA, LRP1B, and SMARCA4 are associated with TMB and play a positive or negative predictive role in ICI treatment (Mehnert et al., 2016; Biton et al., 2018; Li et al., 2018; Chen et al., 2019; Bai et al., 2020; Marinelli et al., 2020; Schoenfeld et al., 2020), none of these studies has explored the potential role of MGA mutation.

In the present study, we attempted to explain the prognostic and predictive role of MGA mutation in ICI treatment of LUAD and other common cancers in detail, and explore the potential mechanisms.

## Materials and Methods

### Patients and Data Sources

**FIGURE 1:**
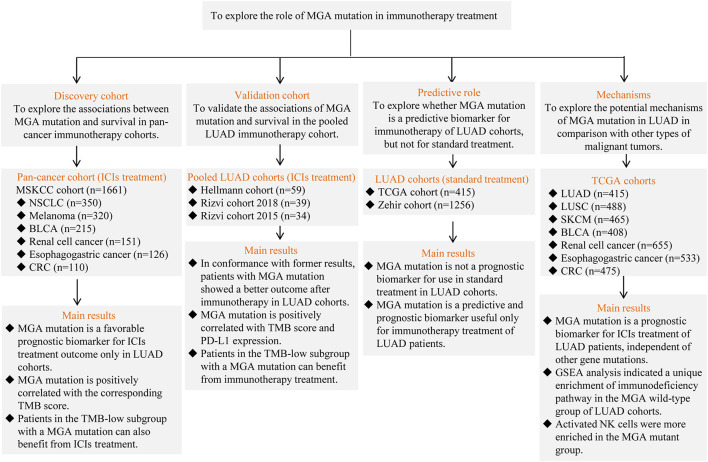
The flow chart of the study. LUAD, lung adenocarcinoma; LUSC, lung squamous cell carcinomas; BLCA, bladder cancer; SKCM, skin cutaneous melanoma; CRC, colorectal cancer; ICIs, immune checkpoint inhibitors; TMB, tumor mutation burden; PD-L1, programmed death-ligand 1; MGA, Max’s Giant Associated protein; TCGA, The Cancer Genome Atlas; GSEA, gene-set enrichment analysis.

The ICI-treatment cohorts analyzed in this study were downloaded from cBioportal^1^ and included the MSKCC cohort (Samstein et al., 2019), Hellmann cohort (Hellmann et al., 2018), Rizvi 2018 cohort (Rizvi et al., 2018), and Rizvi 2015 cohort (Rizvi et al., 2015). A flow diagram explaining the details of each cohort and results is depicted in Figure 1. In brief, we analyzed the data from the pan-cancer MSKCC cohort (discovery cohort; 1,661 patients with malignant tumors treated with ICIs with 468-gene panel sequencing) to explore the potential predictive role of MGA mutation in immunotherapy outcomes. The ICIs used in the MSKCC cohort were anti-PD-1/PD-L1 or a combination of the two. Then, we validated our findings by analyzing a pooled LUAD cohort consisting of three public datasets of patients who received ICI treatment: Hellmann cohort consisting of 59 patients treated with anti-PD-1 plus anti-CTLA-4; Rizvi cohort 2018 (with complete data of TMB score) consisting of 39 patients treated with anti-PD-1 or anti-PD-L1 therapy; and Rizvi cohort 2015 consisting of 34 patients treated with anti-PD-1. A total of 415 LUAD patients from The Cancer Genome Atlas (TCGA)^2^ database and 1,256 LUAD patients from the Zehir cohort in cBioportal (Zehir et al., 2017), who were treated with conventional standard treatment, were analyzed to explore the prognostic value of MGA mutation for non-ICI treatment. We also obtained the Whole exome sequencing (WES) and mRNA expression data of 2,784 pan-cancer data from the TCGA database to elucidate the potential mechanisms underlying the relationship between MGA mutation and immunotherapy.

### Process of Data Quality Inspection

Since public database might include heterogeneity, we firstly performed a data quality inspection to ensure a more rigorous analysis. The process of quality inspection included: (1) Patients with incomplete information of gene mutations, survival, or TMB score were excluded from the analyses; (2) No matter what cut-off value was in the original article, in our analysis, high TMB was considered as patients with top 20% TMB score in the cohorts in accordance to the standard of MSKCC cohort (Samstein et al., 2019). Besides, all the TMB score included in the analysis was detected by WES, and the results detected with MSK-IMPACT panel were excluded from the analysis to minimize the heterogeneity; (3) Similarly, in all cohorts, high expression level of PD-L1 was re-defined as these with ≥ 1% positive PD-L1 expression according to two references (Herbst et al., 2016; Rizvi et al., 2018); (4) For the consistency of the evaluation of treatment outcome, all the cohorts were evaluated with the common evaluation criteria, RECIST V.1.1. In detail, the objective response rate (ORR) was determined as the percentage of patients with complete response (CR) or partial response (PR), and the disease control rate (DCR) was determined as the percentage of patients with complete response (CR), PR, or stable disease (SD). Overall survival (OS) was the time since inception of ICI treatment to the date of death from any cause, whereas progress-free survival (PFS) was defined as the time since the inception of ICI treatment to the date of progressive disease (PD) diagnosis or death from any cause. Patients without disease progression were censored at the date of the last follow-up.

### Gene-Set Enrichment Analysis

GSEA was performed using mRNA sequencing and gene mutation data of pan-cancer patients downloaded from the TCGA database. The results were analyzed using the software GSEA 4.0.2. The pathway map was generated by The KEGG PATHWAY database.^3^

### Analysis of Tumor-Infiltrating Immune Cells

The distribution of 22 types of TIICs were calculated using CIBERSORT with the method developed by Wang et al. (2021). Violin plots were depicted with the package “vioplot” (version 0.3.0).^4^

### Statistical Analyses

Continuous variables were analyzed by the Mann–Whitney *U*-test and categorical variables were analyzed by the χ^2^-test or Fisher’s exact test. Kaplan–Meier curves and log-rank test were used to estimate survival. Variables with a *p*-value < 0.05 in the univariable regression analysis were included to perform multivariable Cox regression analysis. False discovery rate (FDR) was used to evaluate the statistical significance of multi-analyses. *P*-values < 0.05 and FDR *q*-values < 0.25 were considered statistically significant. All analyses and graphs were performed using SPSS software (SPSS 17.0, Chicago, IL, United States), and R V.4.0.4 and GraphPad Prism 5.

## Results

### Tumor Genomic Alterations in the MSKCC Cohort

Data of 1,661 patients in the MSKCC cohort who were treated with ICIs were analyzed. The mutation frequency and mutation type of the top 35 most frequently mutated genes, including MGA, in these patients are presented in Supplementary Figure 1. Among 1,661 cancer patients, 93 (6.6%) of 1,402 profiled samples harbored the MGA mutation, including missense, splice, truncated, and fusion mutations.

### Max’s Giant Associated Protein Mutation Predicted Better Immunotherapeutic Outcomes in Patients With Non-small Cell Lung Cancer

**FIGURE 2:**
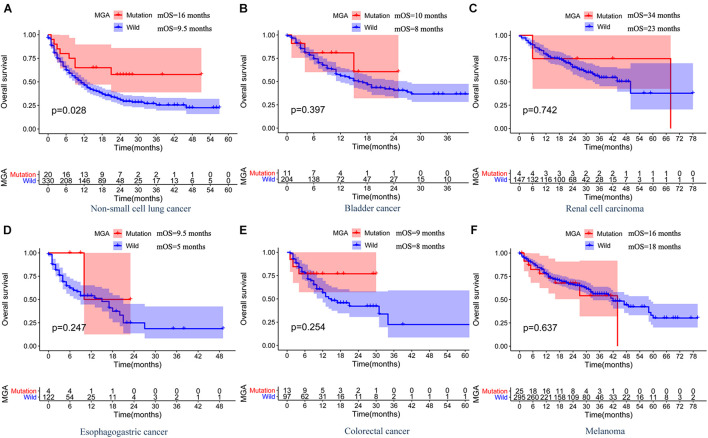
The associations between MGA mutation and OS and in ICI-treated MSKCC pan-cancer cohort. Kaplan-Meier curves of OS between MGA mutant and the wide-type group among patients with NSCLC **(A)**, bladder cancer **(B)**, renal cell carcinoma **(C)**, esophagogastric cancer **(D)**, colorectal cancer **(E)**, and melanoma **(F)**.

**FIGURE 3:**
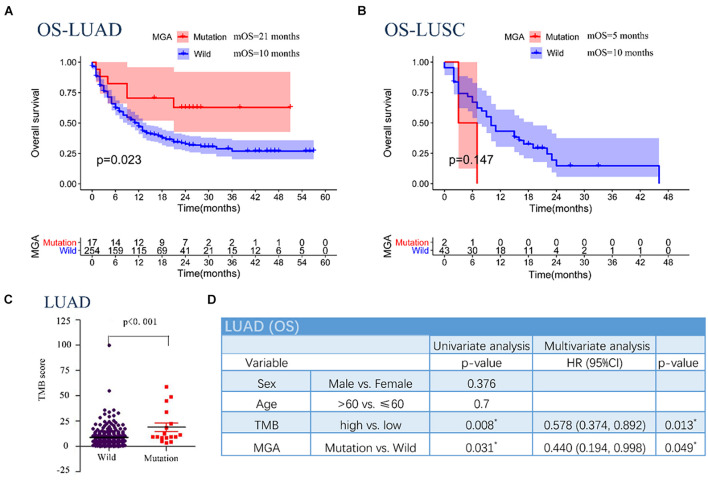
Associations between MGA mutation and OS in different subgroups of pathologic type in patients with NSCLC in the MSKCC pan-cancer cohort. **(A)** Kaplan-Meier survival curves comparing OS between the MGA mutant and wild-type group in the LUAD cohort. **(B)** Kaplan-Meier survival curves comparing OS between the MGA mutant and wild-type group in the LUSC cohort. **(C)** The correlation of MGA mutation and TMB score. **(D)** The univariable and multivariable Cox regression in OS of LUAD patients. *P < 0.05 was considered statistically significant.

According to Kaplan–Meier survival analysis results, MGA mutation was significantly associated with a better immunotherapeutic outcome in the NSCLC cohorts (mOS = 16 months vs. 9.5 months, log-rank test, *p* = 0.028; Figure 2A). However, the associations were not significant in other malignant cancers, including melanoma, bladder cancer, renal cell cancer, esophagogastric cancer, and colorectal cancer (Figures 2B–F). We performed detailed analyses of the data of LUAD and lung squamous cell carcinomas (LUSC) patients in the NSCLC cohorts. Our results showed that MGA mutation was a preferable prognostic factor in LUAD patients but not in LUSC patients (log-rank test, *p* = 0.023, Figure 3A; log-rank test, *p* = 0.147, Figure 3B). LUAD samples with MGA mutation had a higher tumor mutation burden (Wilcoxon rank sum test, *p* < 0.001, Figure 3C). The prognostic value of MGA mutation in LUAD remained statistically significant after considering the TMB score (Cox proportional hazards model, HR, 0.440 (95% CI, 0.194–0.998), *p* = 0.049; Figure 3D). In accordance with the results of the previous studies, our analyses showed that TMB was positive related to prolonged ICI-related OS in LUAD (log-rank test, *p* = 0.006, Supplementary Figure 2A). Interestingly, we also found that in the subgroup with a low TMB score, patients with MGA mutation had better ICI-related OS and the result was almost significant (log-rank test, *p* = 0.054, Supplementary Figure 2B), whereas the association was not significant in the subgroup with a high TMB score (log-rank test, *p* = 0.416, Supplementary Figure 2C). These results indicated that MGA mutation might be a prognostic biomarker only for ICI treatment in LUAD patients and MGA mutation might help optimize patient selection based on TMB score.

### Prognostic Value of Max’s Giant Associated Protein Mutation in the Validation Cohort

**FIGURE 4:**
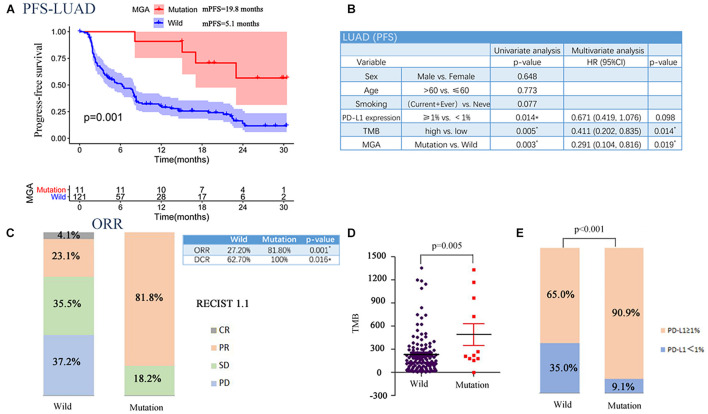
Associations between MGA mutation and PFS in the validation cohort. **(A)** Kaplan-Meier survival curves comparing PFS between the MGA mutant and wild-type group in the LUAD cohort. **(B)** The univariable and multivariable Cox regression in PFS of LUAD patients. **(C)** The ratio of patients with CR, PR, SD, and PD in MGA mutant and wide-type group. **(D)** The correlation of MGA mutation and TMB. **(E)** The correlation of MGA mutation and PD-L1 expression. *P < 0.05 was considered to be statistically significant.

To corroborate the results, we collected WES data from three cohorts that included LUAD patients who had received mono-ICI or dual-ICI treatments. MGA was also found to be frequently mutated [11 of 132 patients (8.3%)] in the pooled LUAD cohort. MGA mutations were significantly associated with better PFS (log-rank test, *p* = 0.001; Figure 4A) even after considering both TMB score and PD-L1 expression status (Cox proportional hazards model, HR, 0.291 (95% CI, 0.104–0.816), *p* = 0.019; Figure 4B). Patients harboring MGA mutation had a higher DCR (100% vs. 62.7%; Fisher’s exact test, *p* = 0.016; Figure 2C) and higher ORR (81.8% vs. 27.2%; Fisher’s exact test, *p* = 0.001; Figure 4C) compared with those with the MGA wild-type. Further analyses showed a positive correlation between MGA mutation and TMB score (Wilcoxon rank sum test, *p* = 0.005, Figure 4D), MGA mutation and PD-L1-positive expression (Fisher’s exact test, *p* < 0.001, Figure 4E) in the aggregated LUAD samples.

**FIGURE 5:**
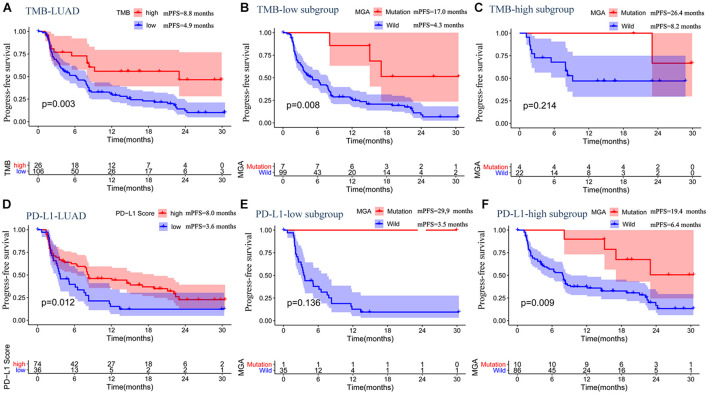
The associations between MGA mutation and PFS in subgroup based on TMB score or PD-L1 expression in the pooled LUAD cohort. **(A)** Kaplan-Meier survival curves comparing PFS between the TMB-low and TMB-high group in the LUAD cohort. **(B)** Kaplan-Meier survival curves comparing PFS between the MGA mutant and wild-type group in the TMB-low subgroup. **(C)** Kaplan-Meier survival curves comparing PFS between the MGA mutant and wild-type group in TMB-high subgroup. **(D)** Kaplan-Meier survival curves comparing PFS between the PD-L1-low group and PD-L1-high group in the LUAD cohort. **(E)** Kaplan-Meier survival curves comparing PFS between the MGA mutant and wild-type group in the PD-L1-low expression score subgroup. **(F)** Kaplan-Meier survival curves comparing PFS between the MGA mutant and wild-type group in the PD-L1-high subgroup.

Similar to the results of the discovery MSKCC cohort, higher TMB and PD-L1-positive expression could predict prolonged ICI-related PFS (Figures 5A,D). Moreover, in subgroup with a low TMB score, patients with MGA mutation had a better ICI-related PFS than those without (log-rank test, *p* = 0.008; Figure 5B), whereas in the subgroup with a TMB score, the result was not significant (log-rank test, *p* = 0.214; Figure 5C). Similarly, MGA mutation predicted favorable ICI-related PFS in the high PD-L1 expression group (log-rank test, *p* = 0.009; Figure 5E), whereas it was not significant in the low PD-L1 expression group (log-rank test, *p* = 0.136; Figure 5F). These results indicate that MGA mutation might help distinguish the responders in TMB-low subgroup.

### Max’s Giant Associated Protein Mutation Was Not a Prognostic Factor for Lung Adenocarcinoma Patients Who Received Standard Treatment

**FIGURE 6:**
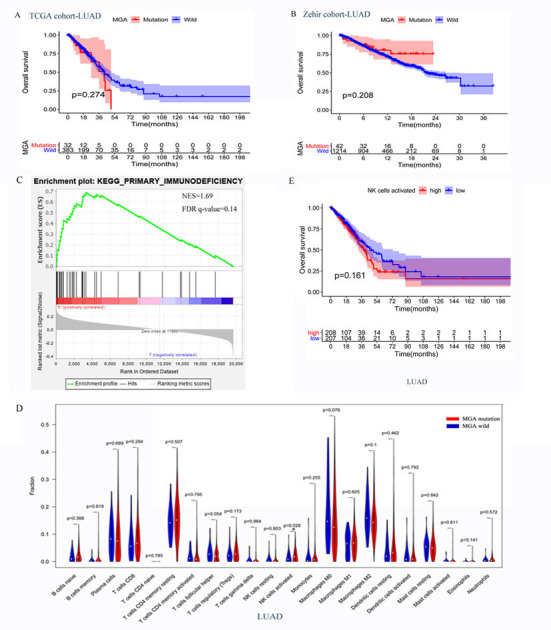
The potential mechanisms of MGA mutation in predicting the outcomes of ICI treatment in LUAD patients. **(A,B)** Associations of MGA mutation and OS in non-ICI treatment cohorts, TCGA cohorts, and Zehir cohorts. **(C)** The enrichment in primary immunodeficiency pathway by Gene Set Enrichment Analysis (GSEA) between MGA mutant and wild-type group in TCGA cohorts. **(D)** Violin plot comparing the TIICs between patients with MGA mutant and wild-type in TCGA cohorts. **(E)** Kaplan-Meier survival curves comparing OS between the activated NK cells low-infiltrating group and high-infiltrating group in the TCGA cohort. ^∗^*P* < 0.05 indicated statistically significant results.

Two LUAD cohorts that included patients who received conventional standard treatment were analyzed to identify whether MGA mutation was a special predictive biomarker for ICI treatment. As shown in Figure 4, Kaplan–Meier survival analyses results showed no significant difference in OS between MGA mutant and wild-type subgroups (415 LUAD patient data from the TCGA database, *p* = 0.274, Figure 6A; 1,256 from the Zehir cohort, *p* = 0.208, Figure 6B), suggesting that MGA mutation was an ICI-specific predictive factor.

### Potential Mechanisms of Max’s Giant Associated Protein Mutation in Predicting the Efficacy of Immune Checkpoint Inhibitor Treatment

**FIGURE 7:**
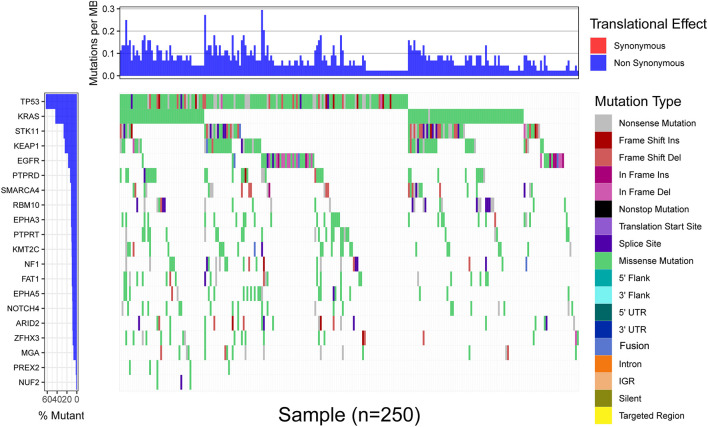
The landscape of the top 20 frequently mutated genes in 271 LUAD patients of the MSKCC cohort. The upper portion is the translational effect, synonymous or non-synonymous. The first row of the lower portion indicate the gene names and the mutant frequency, the second row indicate the mutation type of each mutation in each sample, and the right figure depicts genes mutation types (marked with different colors).

Since MGA mutation played an important role in immunotherapy outcomes, we investigated the potential mechanisms underlying MGA mutation and immune response. The mutation frequency and mutation type of the top 20 frequently mutated genes in the MSKCC LUAD cohort are listed in Figure 7, and the extent of correlations of MGA mutation with the 20 genes is listed in Supplementary Table 1. MGA mutation co-occurred with mutations in only 3 genes (NF1, ZFHX3, and PTPRD) (Fisher’s exact test, *p* < 0.05, *q*-value < 25%), while it did not co-occur with mutations in other genes, including well-known ICI-predictive mutations (e.g., TP53, EGFR, SKT11, and KEAP1). We further investigated whether the co-mutant genes affected the prognostic role of MGA mutation in ICI treatment. Supplementary Figure 3 shows that the relationship between MGA mutation and survival was not affected by NF1, ZFHX3, or PTPRD mutation in LUAD patients, indicating that MGA mutation was an independent prognostic biomarker for ICI treatment. With the mRNA sequencing data of LUAD patients from the TCGA database, GSEA analyses were performed to identify the different enrichment pathways in MGA mutant and wild-type subgroups. Figure 6C show that the immunodeficiency pathway, an important pathway that affects immune cell maturation or function during hematopoiesis (https://www.genome.jp/kegg-bin/show_pathway?hsa05340), was more enriched in the MGA wild-type group (NES = 1.69, FDR *q*-value = 0.14) than the MGA-mutation group. On the other hand, in other malignant tumors, the immunodeficiency pathway was not significantly enriched (Supplementary Figure 4), indicating that MGA mutation might lead to an immune-active status in the LUAD microenvironment. Moreover, we evaluated the abundance of tumor-infiltrating immune cells (TIICs) in the LUAD microenvironment using gene expression data. We found that activated NK cells were more enriched in the MGA-mutation group (*p* = 0.028, Figure 6D) but not in LUSC and other types of malignant tumors (Supplementary Figure 5). Kaplan–Meier survival analysis results showed that activated NK cells were not a prognostic factor for OS in LUAD patients (Figure 6E). These results suggested that the infiltration of activated NK cells might also play an important role in LUAD patients with MGA mutation, thus correlated to the better outcome of ICI treatment.

## Discussion

Our study suggested that MGA mutation could be a promising predictive biomarker for ICI treatment in LUAD patients and could help optimize ICI treatment based on TMB score. In the discovery cohort, among the top 35 mutated genes, five genes (MGA, PBRM1, PTPRD, NOTCH1, ZFHX3) were found to be correlated to ICI outcome in NSCLC patients, while only three genes (MGA, PTPRD, ZFHX3) were significantly associated with patients’ survival in the validation cohorts. In the previous studies, PTPRD/PTPRT mutation and ZFHX3 mutation were reported to be protective biomarker for ICI treatment in NSCLC patients (Bindea et al., 2013; Zhang et al., 2021). However, the detailed role of MGA mutation in the outcome of ICI therapy remains unclear. Therefore, in this study, we attempted to explore the predictive role of MGA mutation in NSCLC patients treated with ICIs.

The classic role of MGA was a TSG that suppressed the progress of tumors by binding to MAX and inhibiting MYC-dependent tumor development (Hurlin et al., 1999). Recently, MGA was found to mutate frequently in multiple types of cancers, thus providing a novel insight into the function of MGA (De Paoli et al., 2013; Cancer Genome, Atlas Research, and Network, 2014; Jo et al., 2016; Zhang et al., 2018). Zengin and Önal-Süzek (2021) reported that MGA was a driver gene in LUAD but not in LUSC. Nonetheless, the specific role of MGA mutation in cancer is unclear, and the role of MGA mutation in ICI treatment outcome was not determined. In this study, we provided statistically significant evidence that MGA mutation was a favorable prognostic biomarker for ICI treatment outcome, which was independent of TMB, PD-L1 expression, and other ICI-related mutations only for patients with LUAD but not for those with LUSC or other common type of cancers. In addition, the prognostic value of MGA mutation is unique for ICI treatment and not for traditional standard treatment. This is the first study to propose and validate MGA mutation as a favorable predictor for ICI treatment in LUAD patients.

To identify the potential mechanisms of MGA mutation participating in the prediction of ICI therapy, GSEA and immune infiltration cells were analyzed between MGA-mutation and wild-type groups. Our results showed that the primary immunodeficiency pathway, which interfered in B and T cell maturation, was significantly up-regulated in the MGA wild-type group compared with the MGA-mutation group, and this significant up-regulation was only observed in LUAD patients but not in those with other types of cancers. In addition, TIIC analysis indicated that activated NK cells were more infiltrated in MGA-mutated samples of LUAD patients. These findings are consistent with those of previous studies that reported that ICI treatment could stimulate NK cells against cancer directly by binding to the inhibitor receptor or indirectly by activating antibody-dependent cell-mediated cytotoxicity (ADCC) (Pesce et al., 2017, 2019, 2020; Segal et al., 2018; Guo et al., 2020). These results might partly explain the predictive role of MGA mutation in ICI treatment in LUAD patients.

Immunotherapy has been widely used in clinical practice, while there is an unmet need in patient selection based on multiple biomarkers. Many existing biomarkers, including PD-L1 expression and TMB score, showed their limitation since they cannot distinguish the potential responsive patients in a perfect way. Across all tumor types, anti-PD-1/PD-L1 therapy results in response rates of 36–100% in patients with PD-L1-positive tumors, whereas in those with PD-L1-negative tumors, response rates range from 0 to 17% (Patel and Kurzrock, 2015). TMB score, independent of PD-L1 expression, was another acknowledged biomarker for ICIs therapy. Whereas as reported by the study from Goodman et al. (2017), 58% patients with high TMB were responsive to immunotherapy, whereas there were also 20% responsive rate observed in those with low and moderate TMB. How to select those responsive patients in PD-L1-low subgroup or TMB-low group remains a problem. The main finding of our analysis was that MGA mutation could be a promising predictive biomarker for ICI treatment. We further performed subgroup analysis to explore whether MGA mutation could help choose responsive patients in PD-L1-low subgroup or TMB-low group. In the subgroup analysis we surprisingly found that MGA mutation could help optimize patient selection based on TMB score. The results indicating that we could select some of these potential responsive patients in these with TMB-low. In the TMB-low patients, who were previously considered to be unresponsive to ICI treatment, these with MGA mutation could also benefit from ICI treatment. While in the subgroup with PD-L1-low expression, MGA mutation status failed to optimize the patient selection. The detailed mechanism need to be clarified in the future. These results might suggest a more personalized use of ICI treatment. One more potential advantage of MGA mutation as a predictive biomarker for ICI treatment is that MGA status might be easily obtained by analyzing peripheral blood samples, thus making it a promising predictive biomarker in the future.

This study had several limitations. First, our study was a retrospective study based on the public database with limited available patients, which might weak the predictive value of MGA mutation. Therefore, a well-designed, prospective, multi-center study with more samples was required to verify the findings. Second, the type of MGA mutation was diverse, including different types of missense, nonsense, and fusion mutations. Limited by the sample size, it was not feasible for our study to clarify the specific type of MGA mutation that was most important in predicting the outcome of ICI treatment. Thus, the specific role of different subtypes of MGA mutation should be considered in a future study. Moreover, our study identified a possible mechanism explaining why MGA status could predict the efficacy of ICI treatment; however, a detailed mechanism of which subtype of MGA mutations needs more proofs from cell line and animal experiments. Third, since both the GSEA analysis and TIIC analysis were performed using data from the TCGA database including patients treated with traditional standard treatment, our analyses could not explain the results that MGA mutation could not predict the prognosis of patients in TCGA database with standard treatment. More studies were required to clarify the mechanisms.

## Data Availability Statement

Publicly available datasets were analyzed in this study. This data can be found here: https://www.cbioportal.org/; https://portal.gdc.cancer.gov/; and https://tcga-data.nci.nih.gov/tcga/.

## Ethics Statement

Ethical review and approval was not required for the study on human participants in accordance with the local legislation and institutional requirements. Written informed consent for participation was not required for this study in accordance with the national legislation and the institutional requirements.

## Author Contributions

JW and HB came up with the idea and design the subject. YQ collected the data, analyzed the data, and wrote the manuscript. CW and LL helped collect and analyze the data. SL, XZ, and ZM raised their suggestion and helped modify the constructure of the manuscript. All authors contributed to the article and approved the submitted version.

## Conflict of Interest

The authors declare that the research was conducted in the absence of any commercial or financial relationships that could be construed as a potential conflict of interest.

## Publisher’s Note

All claims expressed in this article are solely those of the authors and do not necessarily represent those of their affiliated organizations, or those of the publisher, the editors and the reviewers. Any product that may be evaluated in this article, or claim that may be made by its manufacturer, is not guaranteed or endorsed by the publisher.

## Footnotes

1
https://www.cbioportal.org/

2
https://tcga-data.nci.nih.gov/tcga/

3
https://www.genome.jp/kegg-bin/show_pathway?hsa05340

4
https://cran.r-project.org/web/packages/vioplot/index.html

## Supplementary Material

Supplementary Figure 1 The landscape of the top 35 gene mutations in 1,661 pan-cancer patients with ICI treatment. The upper portion is the translational effect, synonymous or non-synonymous. The first row of the lower portion indicate the gene names and the mutant frequency, the second row indicate the mutation type of each mutation in each sample, and the right figure depicts genes mutation types (marked with different colors).

Supplementary Figure 2 Associations between MGA mutation and OS in different subgroups of TMB score in patients with NSCLC in the MSKCC pan-cancer cohort. **(A)** Kaplan-Meier survival curves comparing OS between the low TMB-score group and high TMB-score group in the LUAD cohort. **(B)** Kaplan-Meier survival curves comparing OS between the MGA mutant and wild-type group in the low TMB score subgroup. **(C)** Kaplan-Meier survival curves comparing OS between the MGA mutant and wild-type group in the high TMB score subgroup.

Supplementary Figure 3 The effect of co-mutant genes in the prognostic role of MGA mutation in ICIs treatment in the MSKCC LUAD cohort. The prognostic value of NF1 **(A)**, PTPRD **(B)**, and ZFHX3 **(C)** co-mutant with MGA in ICIs treatment.

Supplementary Figure 4 The enrichment in primary immunodeficiency pathway by GSEA between MGA mutant and wild-type group in the pan-cancer TCGA cohorts. The enrichment in primary immunodeficiency pathway in LUSC **(A)**, skin cutaneous melanoma (SKCM) **(B)**, bladder cancer **(C)**, renal cell carcinoma **(D)**, esophagogastric cancer **(E)**, and colorectal cancer **(F)**.

Supplementary Figure 5 Violin plot comparing the infiltrating immune cells between patients with MGA mutant and wild-type in the pan-cancer TCGA cohorts. Violin plot comparing the infiltrating immune cells between patients with MGA mutant and wild-type in LUSC **(A)**, SKCM **(B)**, bladder cancer **(C)**, renal cell carcinoma **(D)**, esophagogastric cancer **(E)**, and CRC **(F)**.

Supplementary Table 1 Relationship between MGA mutation and the top 20 gene mutations in 271 LUAD patients of the MSKCC cohort. *P* < 0.05 and *q* < 25% were considered to be statistically significant.

## References

[B1] Addeo A. Banna G. L. Weiss G. J. (2019). Tumor mutation burden-from hopes to doubts. *JAMA Oncol.* 5 934–935. 10.1001/jamaoncol.2019.0626 31145420

[B2] Bai H. Duan J. Li C. Xie W. Fang W. Xu Y. (2020). EPHA mutation as a predictor of immunotherapeutic efficacy in lung adenocarcinoma. *J. Immunother. Cancer* 8:e001315. 10.1136/jitc-2020-001315 33303576 PMC7733211

[B3] Bindea G. Mlecnik B. Tosolini M. Kirilovsky A. Waldner M. Obenauf A. C. (2013). Spatiotemporal dynamics of intratumoral immune cells reveal the immune landscape in human cancer. *Immunity* 39 782–795. 24138885 10.1016/j.immuni.2013.10.003

[B4] Biton J. Mansuet-Lupo A. Pécuchet N. Alifano M. Ouakrim H. Arrondeau J. (2018). TP53, STK11, and EGFR mutations predict tumor immune profile and the response to Anti-PD-1 in lung adenocarcinoma. *Clin. Cancer Res.* 24 5710–5723. 10.1158/1078-0432.ccr-18-0163 29764856

[B5] Cancer Genome, Atlas Research, and Network. (2014). Comprehensive molecular profiling of lung adenocarcinoma. *Nature* 511 543–550. 10.1038/nature13385 25079552 PMC4231481

[B6] Chen H. Chong W. Wu Q. Yao Y. Mao M. Wang X. (2019). Association of LRP1B mutation with tumor mutation burden and outcomes in melanoma and non-small cell lung cancer patients treated with immune check-point blockades. *Front. Immunol.* 10:1113. 10.3389/fimmu.2019.01113 PMC6536574 31164891

[B7] Dang C. V. (2012). MYC on the path to cancer. *Cell* 149 22–35. 10.1016/j.cell.2012.03.003 22464321 PMC3345192

[B8] De Paoli L. Cerri M. Monti S. Rasi S. Spina V. Bruscaggin A. (2013). MGA, a suppressor of MYC, is recurrently inactivated in high risk chronic lymphocytic leukemia. *Leukemia Lymphoma* 54 1087–1090. 10.3109/10428194.2012.723706 23039309

[B9] Escudier B. Motzer R. J. Sharma P. Wagstaff J. Plimack E. R. Hammers H. J. (2017). Treatment beyond progression in patients with advanced renal cell carcinoma treated with nivolumab in Checkmate 025. *Eur. Urol.* 72 368–376. 10.1016/j.eururo.2017.03.037 28410865

[B10] Ferris R. L. Blumenschein G. Jr. Fayette J. Guigay J. Colevas A. D. Licitra L. (2016). Nivolumab for recurrent squamous-cell carcinoma of the head and neck. *N. Engl. J. Med.* 375 1856–1867. 27718784 10.1056/NEJMoa1602252 PMC5564292

[B11] Goodman A. M. Kato S. Bazhenova L. Patel S. P. Frampton G. M. Miller V. (2017). Tumor mutational burden as an independent predictor of response to immunotherapy in diverse cancers. *Mol. Cancer Ther.* 16 2598–2608. 10.1158/1535-7163.mct-17-0386 28835386 PMC5670009

[B12] Guo L. Wei R. Lin Y. Kwok H. F. (2020). Clinical and recent patents applications of PD-1/PD-L1 targeting immunotherapy in cancer treatment-current progress, strategy, and future perspective. *Front. Immunol.* 11:1508. 10.3389/fimmu.2020.01508 PMC7358377 32733486

[B13] Hellmann M. D. Nathanson T. Rizvi H. Creelan B. C. Sanchez-Vega F. Ahuja A. (2018). Genomic features of response to combination immunotherapy in patients with advanced non-small-cell lung cancer. *Cancer Cell* 33 843–852. 29657128 10.1016/j.ccell.2018.03.018 PMC5953836

[B14] Herbst R. S. Baas P. Kim D. W. Felip E. Pérez-Gracia J. L. Han J. Y. (2016). Pembrolizumab versus docetaxel for previously treated, PD-L1-positive, advanced non-small-cell lung cancer (KEYNOTE-010): a randomised controlled trial. *Lancet* 387 1540–1550. 10.1016/s0140-6736(15)01281-7 26712084

[B15] Hurlin P. J. Steingrìmsson E. Copeland N. G. Jenkins N. A. Eisenman R. N. (1999). Mga, a dual-specificity transcription factor that interacts with Max and contains a T-domain DNA-binding motif. *EMBO J.* 18 7019–7028. 10.1093/emboj/18.24.7019 10601024 PMC1171765

[B16] Jo Y. S. Kim M. S. Yoo N. J. Lee S. H. (2016). Somatic mutation of a candidate tumour suppressor MGA gene and its mutational heterogeneity in colorectal cancers. *Pathology* 48 525–527. 10.1016/j.pathol.2016.04.010 27306572

[B17] Li X. Pasche B. Zhang W. Chen K. (2018). Association of MUC16 mutation with tumor mutation load and outcomes in patients with gastric cancer. *JAMA Oncol.* 4 1691–1698. 10.1001/jamaoncol.2018.2805 30098163 PMC6440715

[B18] Marinelli D. Mazzotta M. Scalera S. Terrenato I. Sperati F. D’Ambrosio L. (2020). KEAP1-driven co-mutations in lung adenocarcinoma unresponsive to immunotherapy despite high tumor mutational burden. *Ann. Oncol.* 31 1746–1754. 10.1016/j.annonc.2020.08.2105 32866624

[B19] Mehnert J. M. Panda A. Zhong H. Hirshfield K. Damare S. Lane K. (2016). Immune activation and response to pembrolizumab in POLE-mutant endometrial cancer. *J. Clin. Invest.* 126 2334–2340. 10.1172/jci84940 27159395 PMC4887167

[B20] Patel S. P. Kurzrock R. (2015). PD-L1 expression as a predictive biomarker in cancer immunotherapy. *Mol. Cancer Ther.* 14 847–856. 10.1158/1535-7163.mct-14-0983 25695955

[B21] Pesce S. Belgrano V. Greppi M. Carlomagno S. Squillario M. Barla A. (2019). Different features of tumor-associated NK cells in patients with low-grade or high-grade peritoneal carcinomatosis. *Front. Immunol.* 10:1963. 10.3389/fimmu.2019.01963 PMC6712073 31497016

[B22] Pesce S. Greppi M. Tabellini G. Rampinelli F. Parolini S. Olive D. (2017). Identification of a subset of human natural killer cells expressing high levels of programmed death 1: a phenotypic and functional characterization. *J. Allergy Clin. Immunol.* 139 335–346.e333. 27372564 10.1016/j.jaci.2016.04.025

[B23] Pesce S. Trabanelli S. Di Vito C. (2020). Cancer immunotherapy by blocking immune checkpoints on innate lymphocytes. *Cancers* 12:3504. 10.3390/cancers12123504 33255582 PMC7760325

[B24] Remon J. Passiglia F. Ahn M. J. Barlesi F. Forde P. M. Garon E. B. (2020). Immune checkpoint inhibitors in thoracic malignancies: review of the existing evidence by an IASLC expert panel and recommendations. *J. Thoracic Oncol.* 15 914–947. 10.1016/j.jtho.2020.03.006 32179179

[B25] Rizvi H. Sanchez-Vega F. La K. Chatila W. Jonsson P. Halpenny D. (2018). Molecular determinants of response to anti-programmed cell death (PD)-1 and anti-programmed death-ligand 1 (PD-L1) blockade in patients with non-small-cell lung cancer profiled with targeted next-generation sequencing. *J. Clin. Oncol.* 36 633–641. 29337640 10.1200/JCO.2017.75.3384 PMC6075848

[B26] Rizvi N. A. Hellmann M. D. Snyder A. Kvistborg P. Makarov V. Havel J. J. (2015). Cancer immunology. mutational landscape determines sensitivity to PD-1 blockade in non-small cell lung cancer. *Science* 348 124–128. 25765070 10.1126/science.aaa1348 PMC4993154

[B27] Samstein R. M. Lee C. H. Shoushtari A. N. Hellmann M. D. Shen R. Janjigian Y. Y. (2019). Tumor mutational load predicts survival after immunotherapy across multiple cancer types. *Nat. Genet.* 51 202–206. 30643254 10.1038/s41588-018-0312-8 PMC6365097

[B28] Schoenfeld A. J. Bandlamudi C. Lavery J. A. (2020). The genomic landscape of SMARCA4 alterations and associations with outcomes in patients with lung cancer. *Clin. Cancer Res.* 26 5701–5708. 10.1158/1078-0432.ccr-20-1825 32709715 PMC7641983

[B29] Segal N. H. Naidoo J. Curigliano G. Patel S. Diamond J. R. (2018). First-in-human dose escalation of monalizumab plus durvalumab, with expansion in patients with metastatic microsatellite-stable colorectal cancer. *J. Clin. Oncol.* 36:3540. 10.1200/jco.2018.36.15_suppl.3540

[B30] Wang X. Wu B. Yan Z. Wang G. Chen S. Zeng J. (2021). Association of PTPRD/PTPRT mutation with better clinical outcomes in NSCLC patients treated with immune checkpoint blockades. *Front. Oncol.* 11:650122. 10.3389/fonc.2021.650122 PMC8192300 34123798

[B31] Wolchok J. D. Chiarion-Sileni V. Gonzalez R. Rutkowski P. Grob J. J. Cowey C. L. (2017). Overall survival with combined nivolumab and ipilimumab in advanced melanoma. *N. Engl. J. Med.* 377 1345–1356. 28889792 10.1056/NEJMoa1709684 PMC5706778

[B32] Wu Y. L. Lu S. Cheng Y. Zhou C. Wang J. Mok T. (2019). Nivolumab versus docetaxel in a predominantly chinese patient population with previously treated advanced NSCLC: CheckMate 078 randomized phase III clinical trial. *J. Thorac. Oncol.* 14 867–875. 10.1016/j.jtho.2019.01.006 30659987

[B33] Yang X. Yin R. Xu L. (2018). Neoadjuvant PD-1 blockade in resectable lung cancer. *N. Engl. J. Med.* 379:e14. 10.1056/nejmc1808251 30179394

[B34] Zehir A. Benayed R. Shah R. H. Syed A. Middha S. (2017). Mutational landscape of metastatic cancer revealed from prospective clinical sequencing of 10,000 patients. *Nat. Med.* 23 703–713. 28481359 10.1038/nm.4333 PMC5461196

[B35] Zengin T. Önal-Süzek T. (2021). Comprehensive profiling of genomic and transcriptomic differences between risk groups of lung adenocarcinoma and lung squamous cell Carcinoma. *J. Pers. Med.* 11:154. 10.3390/jpm11020154 33672117 PMC7926392

[B36] Zhang J. Zhou N. Lin A. Luo P. Chen X. Deng H. (2021). ZFHX3 mutation as a protective biomarker for immune checkpoint blockade in non-small cell lung cancer. *Cancer Immunol. Immunother.* 70 137–151. 10.1007/s00262-020-02668-8 32653938 PMC10992006

[B37] Zhang Y. Li C. Xue W. Zhang M. Li Z. (2018). Frequent mutations in natural killer/T cell lymphoma. *Cellular Physiol. Biochem.* 49 1–16. 10.1159/000492835 30134235

