# Supplementary figures and images for: Predictive Value of Max’s Giant Associated Protein Mutation in Outcomes of Lung Adenocarcinoma Patients Treated With Immune Checkpoint Inhibitors
Source: Front Cell Dev Biol. 2021 Oct 18;9:728647. doi: 10.3389/fcell.2021.728647 (PMC8558674; doi:10.3389/fcell.2021.728647)

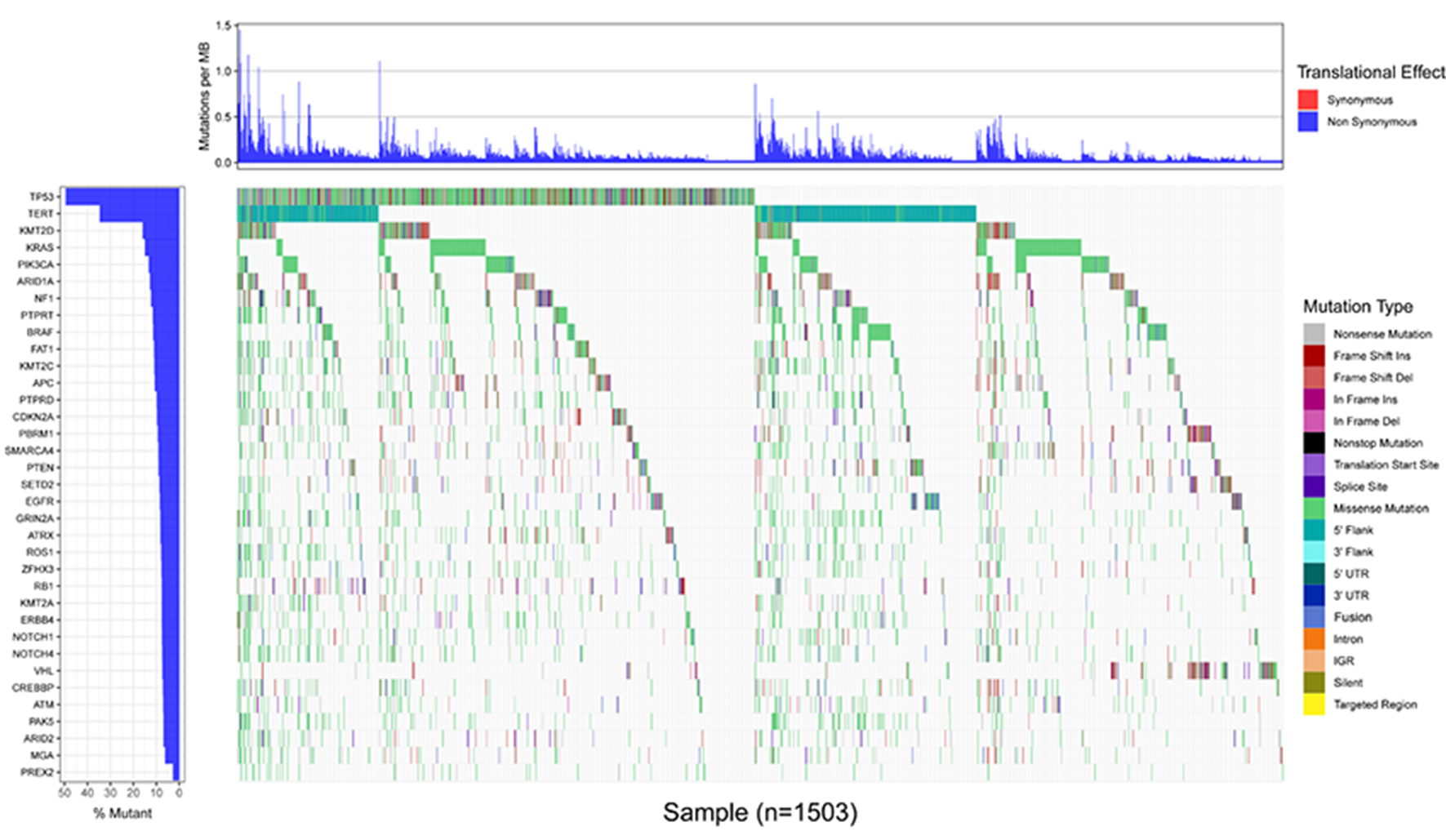

Supplement: Supplementary Figure 1 — The landscape of the top 35 gene mutations in 1,661 pan-cancer patients with ICI treatment. The upper portion is the translational effect, synonymous or non-synonymous. The first row of the lower portion indicate the gene names and the mutant frequency, the second row indicate the mutation type of each mutation in each sample, and the right figure depicts genes mutation types (marked with different colors). [file Image_1.TIF]

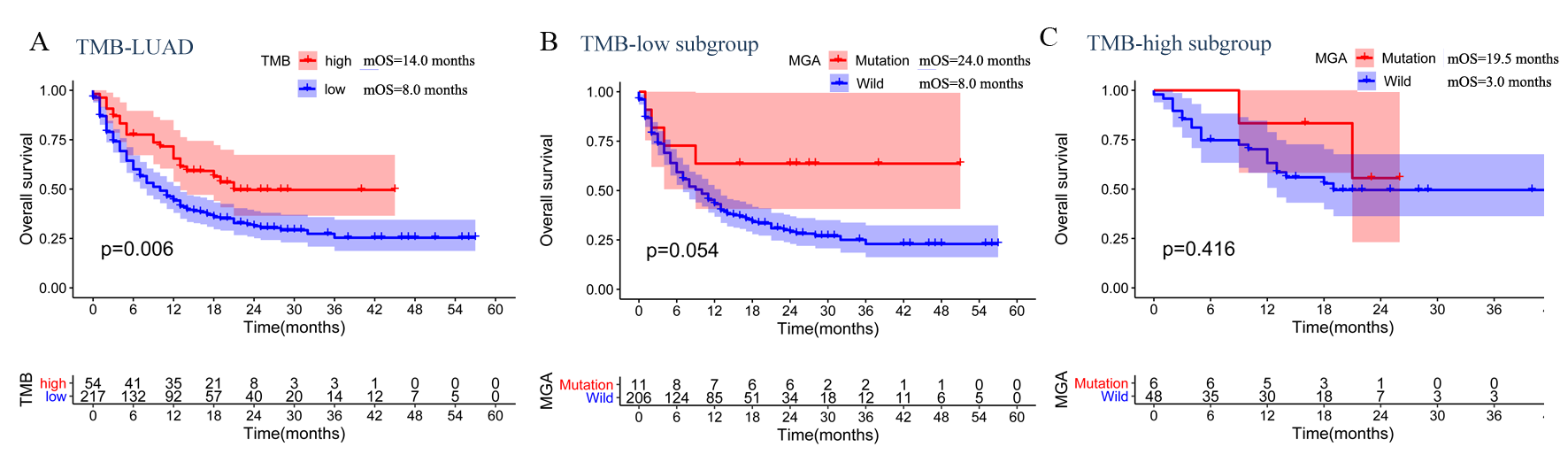

Supplement: Supplementary Figure 2 — Associations between MGA mutation and OS in different subgroups of TMB score in patients with NSCLC in the MSKCC pan-cancer cohort. (A) Kaplan-Meier survival curves comparing OS between the low TMB-score group and high TMB-score group in the LUAD cohort. (B) Kaplan-Meier survival curves comparing OS between the MGA mutant and wild-type group in the low TMB score subgroup. (C) Kaplan-Meier survival curves comparing OS between the MGA mutant and wild-type group in the high TMB score subgroup. [file Image_2.TIF]

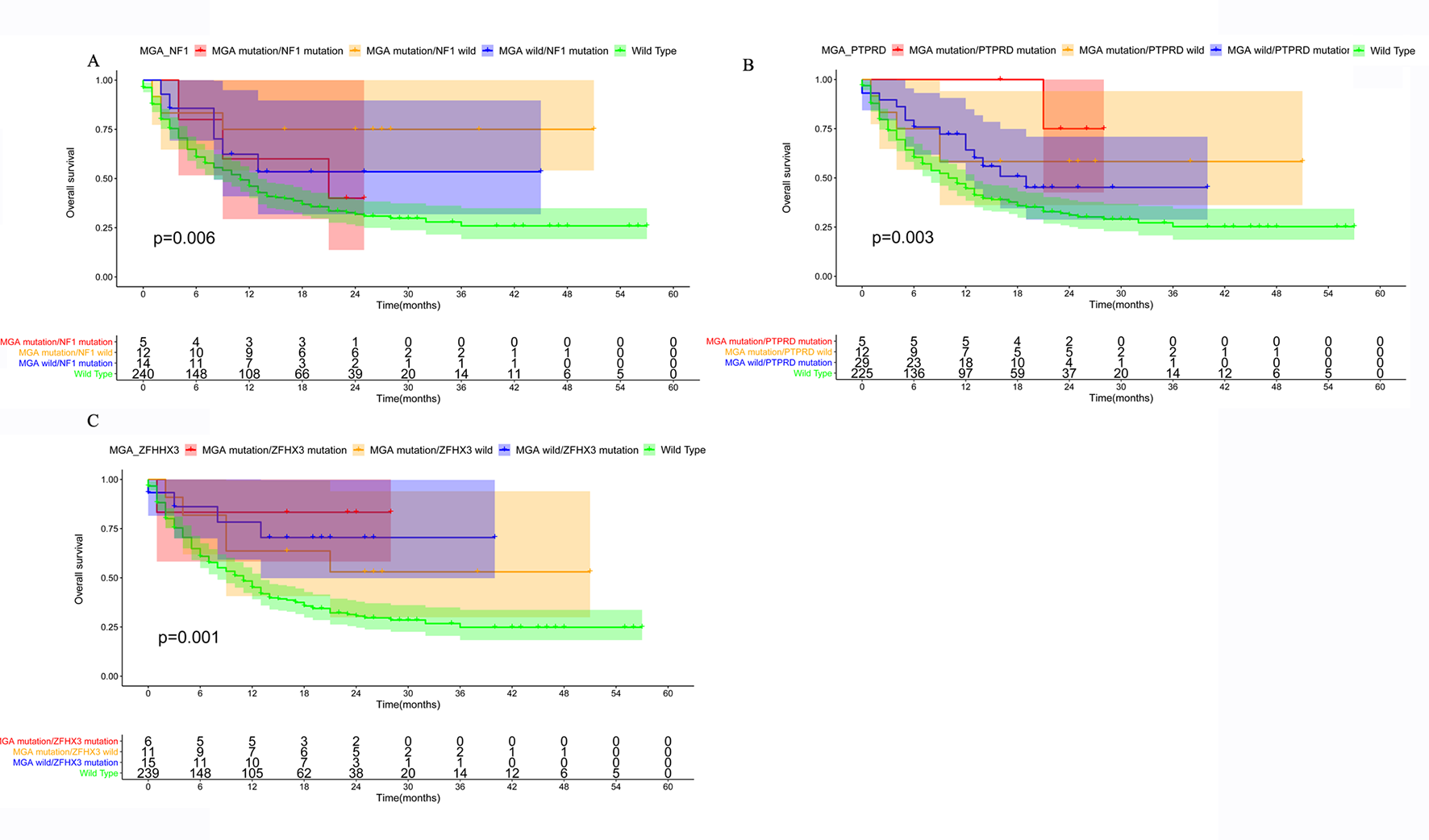

Supplement: Supplementary Figure 3 — The effect of co-mutant genes in the prognostic role of MGA mutation in ICIs treatment in the MSKCC LUAD cohort. The prognostic value of NF1 (A), PTPRD (B), and ZFHX3 (C) co-mutant with MGA in ICIs treatment. [file Image_3.TIF]

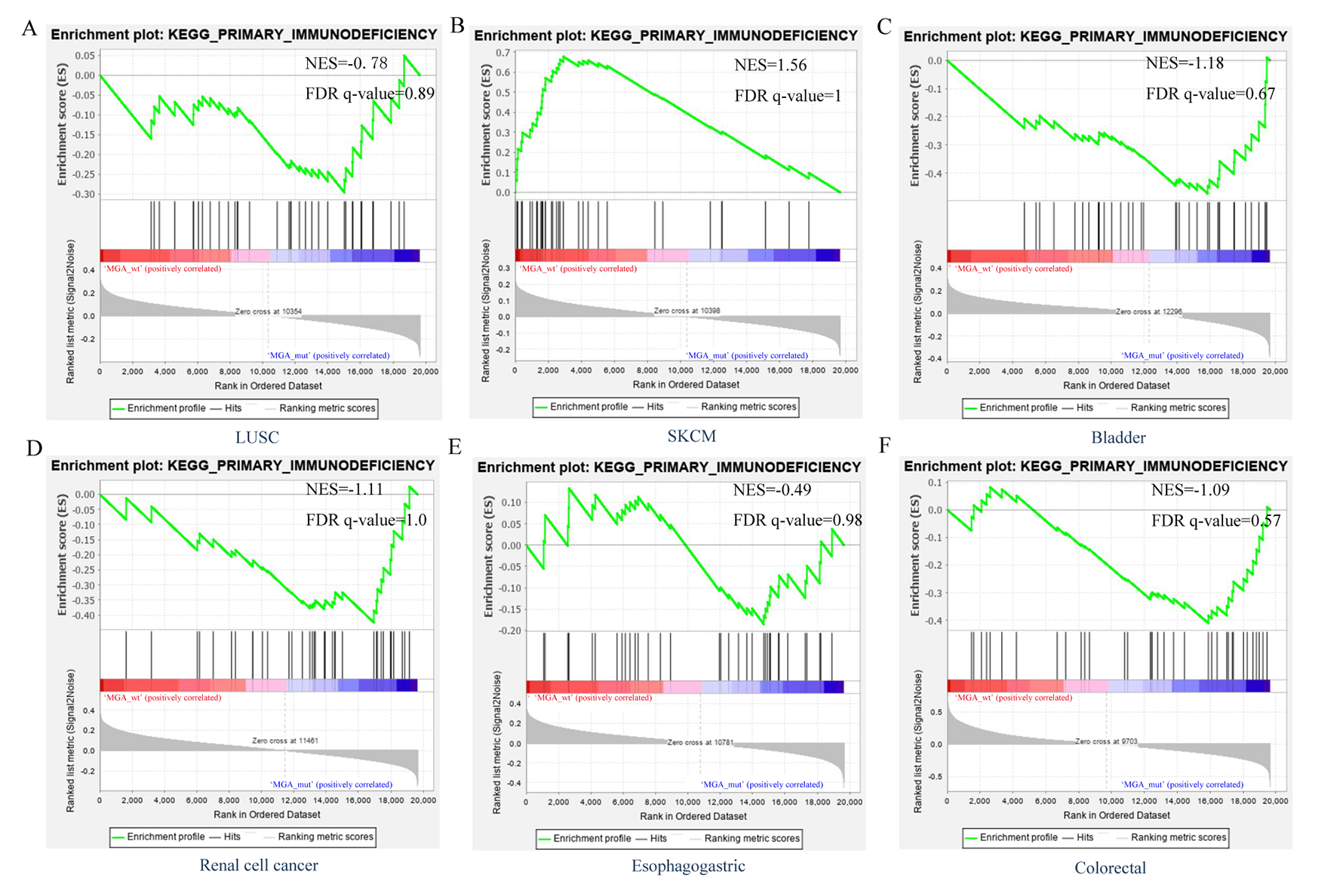

Supplement: Supplementary Figure 4 — The enrichment in primary immunodeficiency pathway by GSEA between MGA mutant and wild-type group in the pan-cancer TCGA cohorts. The enrichment in primary immunodeficiency pathway in LUSC (A), skin cutaneous melanoma (SKCM) (B), bladder cancer (C), renal cell carcinoma (D), esophagogastric cancer (E), and colorectal cancer (F). [file Image_4.TIF]

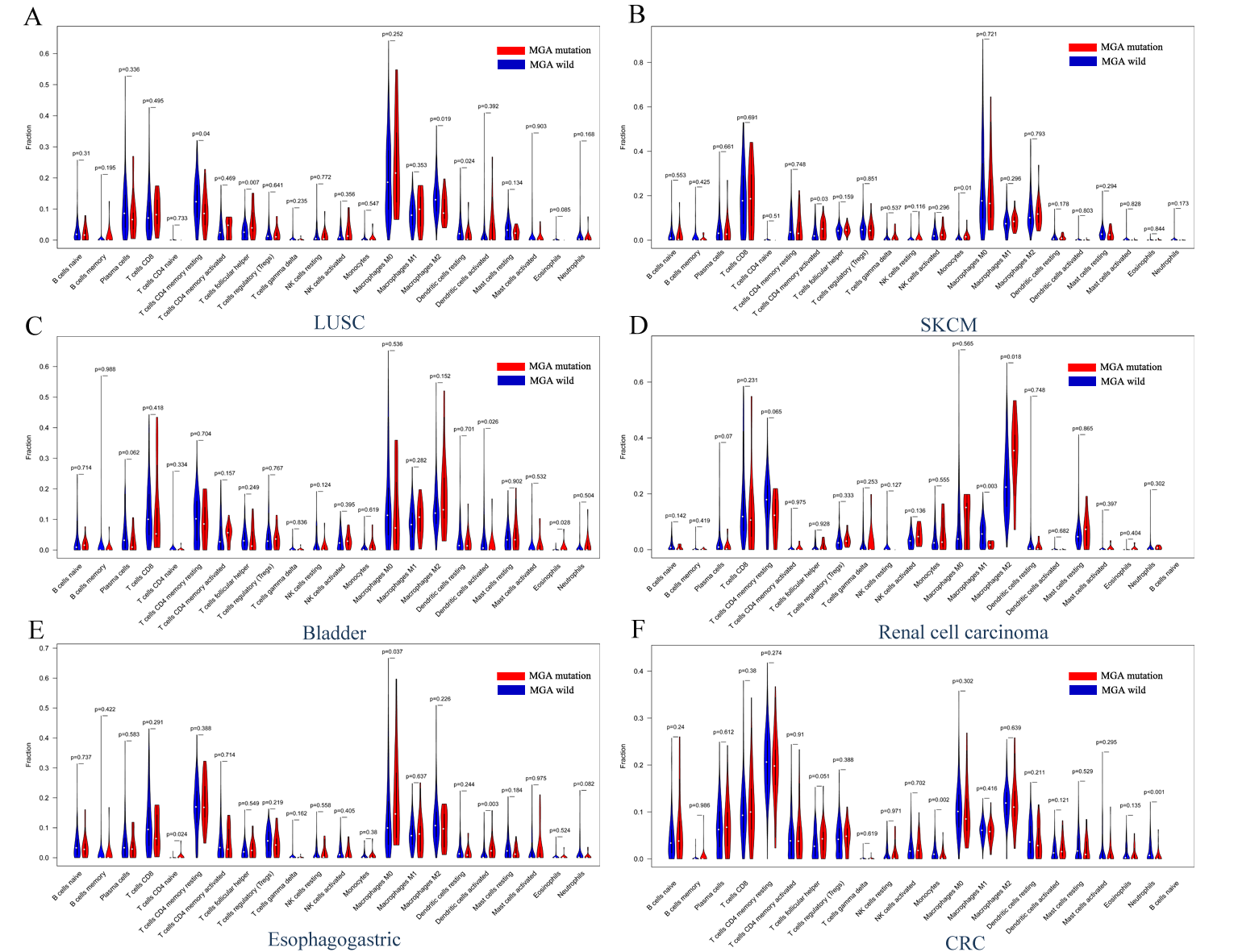

Supplement: Supplementary Figure 5 — Violin plot comparing the infiltrating immune cells between patients with MGA mutant and wild-type in the pan-cancer TCGA cohorts. Violin plot comparing the infiltrating immune cells between patients with MGA mutant and wild-type in LUSC (A), SKCM (B), bladder cancer (C), renal cell carcinoma (D), esophagogastric cancer (E), and CRC (F). [file Image_5.TIF]

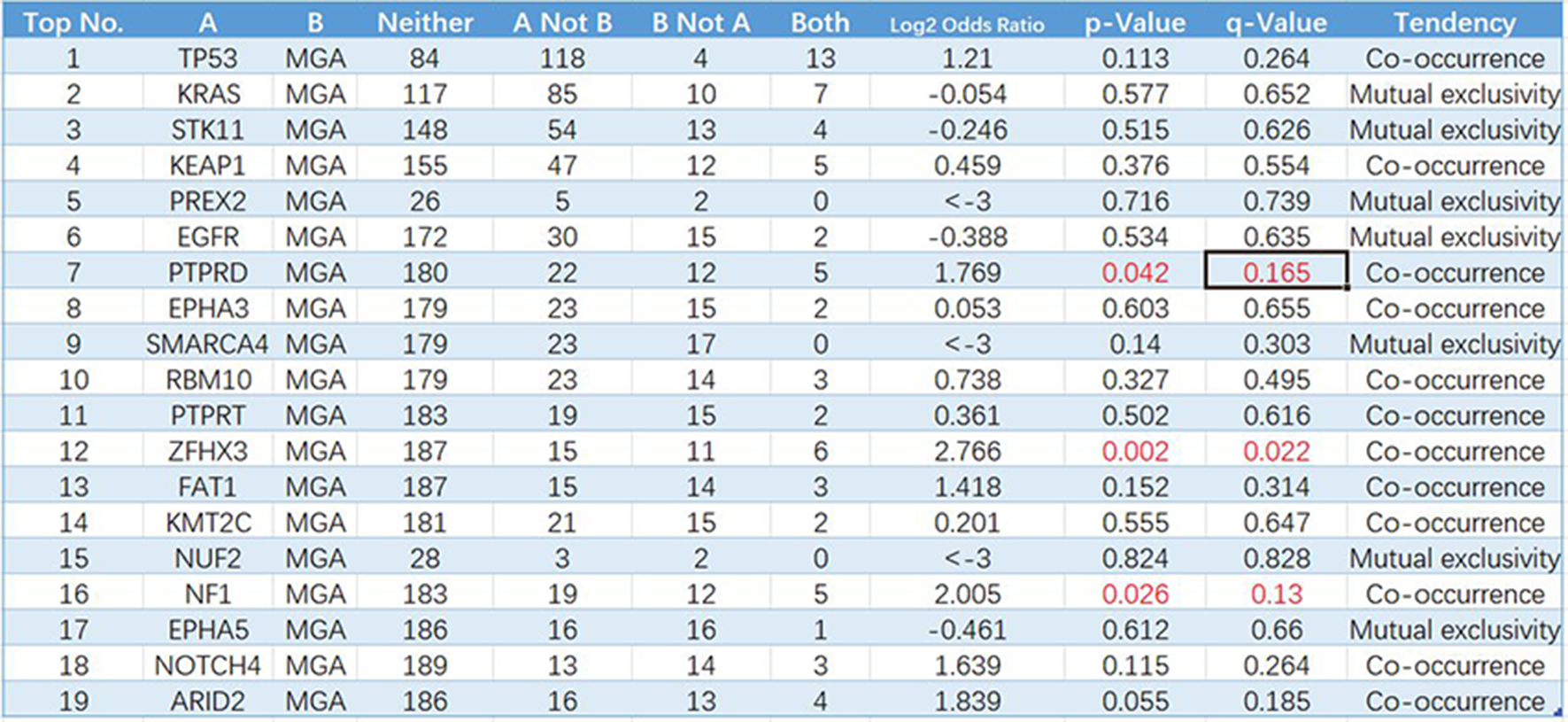

Supplement: Supplementary Table 1 — Relationship between MGA mutation and the top 20 gene mutations in 271 LUAD patients of the MSKCC cohort. P < 0.05 and q < 25% were considered to be statistically significant. [file Image_6.TIF]
